# Establishment and multifaceted characterization of a graded spinal cord injury model based on graduated impact depth

**DOI:** 10.1002/ame2.70150

**Published:** 2026-02-27

**Authors:** Gang Zhou, Yue Hu, Zhimin Li, Shiyuan Han, Yuejia Li, Yongning Li, Jun Gao

**Affiliations:** ^1^ Department of Neurosurgery Peking Union Medical College Hospital, Chinese Academy of Medical Sciences & Peking Union Medical College Beijing China; ^2^ Department of International Medical Service Peking Union Medical College Hospital, Chinese Academy of Medical Sciences & Peking Union Medical College Beijing China

**Keywords:** graded spinal cord injury, histology, locomotor function, magnetic resonance imaging, transcriptome profile

## Abstract

**Background:**

Contusion spinal cord injury (SCI) models are extensively used in preclinical research because of their ability to mimic the pathophysiological characteristics observed in humans. Although various impact devices have been developed to establish graded contusion SCI models, few studies have systematically investigated the relationship between impact depth and injury severity. In this study, we aimed to establish and characterize a graded SCI model, with impact depth as the independent variable.

**Methods:**

A precise impactor system was used to establish graded SCI models by adjusting the impact depth, with comprehensive evaluations of locomotor function, imaging, histology, and transcriptomic profiles of the injured spinal cord. Additionally, gene expression trend analysis and subsequent Gene Ontology enrichment analysis were performed to investigate the potential biological mechanisms associated with injury severity.

**Results:**

The Basso, Beattie, Bresnahan (BBB) score and CatWalk gait analysis demonstrated a severity‐dependent functional recovery pattern in different impact depth groups across multiple postinjury time points. Magnetic resonance imaging and histological results revealed a correlation between impact depth and lesion size. Principal component analysis and heat map clustering of the transcriptomic profile revealed intragroup clustering and intergroup separation, with different injury severities across different time points postinjury. Furthermore, biological mechanisms that correlated with injury severity were identified using gene expression trend analysis.

**Conclusions:**

This study established a quantifiable, graded rat SCI model that was comprehensively evaluated using multiple approaches and may serve as a valuable platform for future SCI research.

## Graphical abstract

We established a graded spinal cord injury (SCI) model based on graduated impact depth, which was subsequently characterized across multiple dimensions, including locomotor function, imaging, histology, and transcriptomic profiles. We further identified transcriptomic changes in graded SCI at different time points postinjury and investigated the biological mechanisms associated with injury severity.

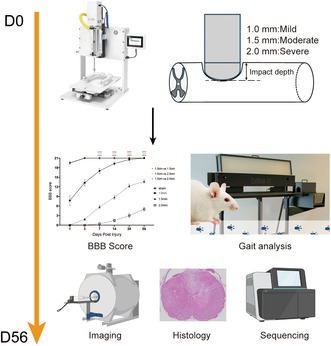

## 1 INTRODUCTION

Preclinical rodent models are essential for the investigation of the pathophysiological mechanisms and regenerative strategies of spinal cord injury (SCI).^1^ In clinical settings, a substantial proportion of SCI cases are anatomically incomplete injuries of varying severities, each demonstrating unique recovery trajectories and necessitating personalized therapeutic strategies.^2^, ^3^ This heterogeneity underscores the need for graded SCI models that can recapitulate the spectrum of human injuries. Two primary approaches have been developed to establish graded SCI in rodents: crush injury with forceps^4^ and contusion injury with impactors.^5^, ^6^, ^7^ However, these models exhibit variations in the pathophysiological process of SCI, which pose challenges for cross‐study comparisons.^8^ In humans, SCIs predominantly result from physical trauma that induces a sudden impact and subsequent compression.^9^, ^10^ Consequently, contusion models can better replicate the biomechanics of clinical SCI. Most graded contusion injury models have been established using the Infinite Horizon impactor with different forces.^5^, ^11^, ^12^ However, a key limitation of this system is that tissue displacement, an important determinant of postinjury outcomes, cannot be directly controlled.^5^, ^13^ Moreover, most existing models are characterized only using locomotor assessments and histological results. Comprehensive imaging and transcriptomic‐level validation on these models are lacking. These limitations highlight the unmet need for a quantitatively graded SCI model that allows the precise control of tissue displacement and integrates multidimensional evaluation methods.

In this study, we aimed to establish a graded SCI model using a precise impactor device (Figure S1A). We directly controlled tissue displacement by manipulating the impact depth, thereby inducing reproducible graded injuries. To validate the model, we conducted multifaceted assessments across behavioral, imaging, histological, and transcriptomic levels. Finally, we explored the biological mechanisms associated with injury severities. Overall, our study established a quantifiable and graded rat SCI model that may be a valuable tool for future research.

## 2 METHODS

### 2.1 Animals and study design

**FIGURE 1:**
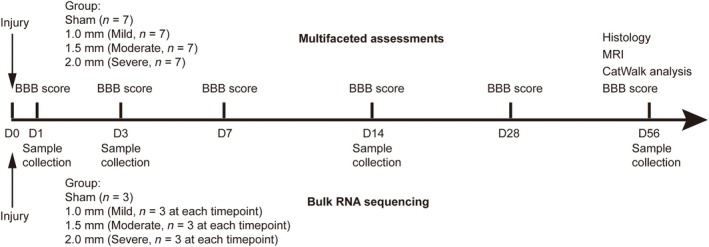
A time‐line diagram of the study design and workflow.

A total of 72 male Sprague–Dawley rats (200–220 g, 9 weeks old, SPF [Beijing] Biotechnology Co., Ltd., Beijing, China) were used in this study. For modeling and assessment, 28 rats were randomly assigned to the sham (*n* = 7), 1.0 mm (*n* = 7), 1.5 mm (*n* = 7), and 2.0 mm (*n* = 7) groups. Two rats in the 2.0‐mm group died after surgery and were replaced with additional models to maintain the group size. For bulk RNA sequencing, three rats were assigned for sample collection for each injury severity at 1, 3, 14, and 56 day postinjury (dpi). Three rats in the 2.0‐mm group died after surgery and were replaced with replicates. Three rats were assigned to the sham group for sequencing. All animal experimental procedures were conducted in the Animal Experiment Center of Peking Union Medical College Hospital and were approved by the Animal Ethics Committee (approval number: XHDW‐2024‐74). The workflow is shown in Figure 1.

### 2.2 Establishment of SCI models

The rats were anesthetized with an intraperitoneal injection of tribromoethanol solution (1.25%, 10 mL/kg). Then, single laminectomy was performed at T9, which was identified based on the projection direction of the spinous processes,^6^, ^14^ to expose the spinal cord. The injury models were established according to the manufacturer's protocol for the impactor (68099 II Precise Impactor, RWD). Briefly, the vertebral column was stabilized using a spinal adaptor by clamping the bilateral sides at T9 level, after which the rat was securely positioned on the impactor (Figure S1B). The adaptor base was adjusted to ensure that the surface of the spinal cord remained horizontal and that the central cord vessel was precisely aligned beneath the center of the impactor tip (Figure S1B). The impact tip had a smooth end with a diameter of 3.0 mm. The impact velocity was set at 3 m/s, with a dwell time of 5 s. After successful zero‐point calibration, SCI was induced at three distinct severities based on impact depth: mild (1.0 mm), moderate (1.5 mm), and severe (2.0 mm). The impact depth was defined as the vertical displacement of the impactor tip from the dorsal surface of the spinal cord midline (Figure S1C). The presence of a subdural hematoma at the injury site, with or without accompanying involuntary tail flicks or intermittent hindlimb spasms, was considered indicative of a successful injury. The sham group underwent T9 laminectomy without injury. Postoperative care included subcutaneous injections of buprenorphine (0.05 mg/kg) twice daily for 3 days to manage pain, along with an intramuscular injection of cefazolin (33.3 mg/kg, twice daily) for 5 days to prevent infection. Manual bladder expression was performed twice daily until autonomous urinary function was restored.

### 2.3 Locomotor assessment

Two evaluation paradigms were used to assess the locomotor function after SCI. First, the Basso, Beattie, Bresnahan (BBB) locomotor rating scale was used to assess open‐field motor function across the groups. Each rat was placed in an open field and assessed for hindlimb locomotor function at 1, 3, 7, 14, 28, and 56 dpi. The assessment was performed independently by two blinded observers, and the final score for each rat was determined by averaging the scores. To further evaluate locomotive function, gait analysis was conducted using the CatWalk XT system (Noldus, version 10.6) at 56 dpi. Three rats from each group that had similar body weights and walked smoothly through the runway were selected for gait analysis. The recorded signals were manually categorized and labeled to identify the paws (right/left forelimbs and hind limbs) and remove other parts of the body (nose, abdomen, and tail) and artifacts. Key gait parameters (Table S1) were extracted from the final dataset for further analysis.^15^, ^16^

### 2.4 Magnetic resonance imaging

All rats underwent magnetic resonance imaging (MRI) in a 7.0‐T small animal system (Bruker BioSpec70/20USR, Germany) at 56 dpi. Anesthesia was initiated with 4%–5% isoflurane and sustained at a concentration of 1.5%–2.5% throughout the scanning process. For imaging, the rats were positioned prone, with the T9 spinal segment centered on a four‐channel surface coil for receiving signals, and an 86‐mm volume coil was used to transmit signals. A breathing sensor was secured to the abdomen, maintaining a controlled range of 40–70 breaths per minute to optimize animal welfare and image quality. Axial, multi‐echo, T2‐weighted rapid acquisition with relaxation enhancement images, with 0.8‐mm slice thickness, 40 × 40‐mm field of view, and a 256 × 256 matrix, were centered on the injury site, with a repetition time of 999 ms and echo times of 28 ms. The Digital Imaging and Communications in Medicine (DICOM) documents were processed using three‐dimensional (3D) Slicer software (version 5.2.0). The area of T2 hyperintensity in the spinal cord was defined as the lesion area, including the central cavity and surrounding scars.^17^, ^18^ The lesion area was segmented on each axial slide and reconstructed as a 3D lesion region. The surface areas and volumes of the lesions were calculated for further analysis.

### 2.5 Spinal cord tissue processing

All rats were killed at 56 dpi with pentobarbital and perfused with phosphate‐buffered saline followed by 4% paraformaldehyde (PFA). Furthermore, the injured spinal cord was obtained and fixed in 4% PFA at 4°C overnight. Then, five samples from each group were sequentially dehydrated in graded ethanol solutions, cleared with xylene, and embedded in paraffin. These samples were then cut into axial sections (10 μm thickness) using a microtome. The remaining two samples in each group were dehydrated in 30% sucrose solution, embedded in OCT compound, and cut into sagittal sections (30 μm thickness) using a cryostat. Slices surrounding the center of the injury site were selected for further histological analysis. Axial sections were stained with hematoxylin–eosin (HE) and Luxol Fast Blue (LFB), whereas sagittal sections were used for immunofluorescence staining to detect glial fibrillary acidic protein (GFAP), neuronal nuclei (NeuN), ionized calcium‐binding adapter molecule 1 (IBA1), and oligodendrocyte transcription factor 2 (Olig2). Details of histological staining are described in Supporting Information.

### 2.6 Principal component analysis and heat map clustering

Details of sample collection, RNA extraction, clustering, and sequencing are described in Supporting Information. Sequencing and assembly information obtained from Illumina sequencing is provided in Table S2. Principal component analysis (PCA) was performed using the prcomp function in R and visualized using the *ggplot2* package to evaluate sample distribution. For hierarchical clustering and heat map visualization, genes with Fragments Per Kilobase of transcript per million (FPKM) >1 in at least three samples were selected. The variance of these genes was calculated, and the 200 most variable genes were retained for further analysis. The FPKM values were standardized using *Z*‐score normalization before clustering. Hierarchical clustering was performed using the *pheatmap* package.

### 2.7 Gene set enrichment analysis and Gene Ontology enrichment analysis

To determine whether the general biological processes of SCI were activated in each injury group, we conducted gene set enrichment analysis (GSEA) using the *clusterProfiler* package to assess the enrichment of predefined gene sets under different experimental conditions.^19^ Certain generally recognized biological processes in the acute, subacute, intermediate, and chronic phases of SCI were selected for testing (Table 1). To compare the differences in biological processes in each severity group, we performed Gene Ontology (GO) enrichment analysis of differentially expressed genes (DEG) using the *GOseq* package.^20^ The top 15 terms enriched in biological processes were selected for comparison and visualization using *ggplot2* and *enrichplot* packages.

**TABLE 1:** Biological process selected for GSEA.

Time point (dpi)	Selected biological process after SCI
1	Acute inflammatory response, neuron apoptotic process, neutrophil migration, response to hypoxia, response to mechanical stimulus
3	Cellular response to oxidative stress, microglia cell activation, phagocytosis, regulation of inflammatory response, regulation of macrophage migration
14	Extracellular matrix organization, extracellular structure organization, glia cell activation, neuroinflammatory response, wound healing
56	Collagen fibril organization, axon ensheathment in central nervous system, regeneration, tissue remolding, wound healing

Abbreviations: dpi, day postinjury; GSEA, gene set enrichment analysis; SCI, spinal cord injury.

### 2.8 Gene expression trend analysis

To investigate the dynamic gene expression patterns across different injury severity groups, we used the *Mfuzz* package for soft clustering–based expression trend analysis.^21^ This method enabled the identification of co‐expressed gene clusters and facilitated the exploration of temporal expression trajectories associated with SCI. Gene clusters representing distinct expression trajectories were visualized using the mfuzz.plot function. Representative clusters exhibiting consistent upregulation or downregulation in injury severity were identified. GO enrichment analysis was performed on these clustered genes, and the top three results for biological processes, cellular components, and molecular functions were selected for analysis.

### 2.9 Statistical analysis

Data were presented as mean ± standard error of the mean. Data visualization and statistical analyses were performed using GraphPad Prism (version 8.30). The normality of datasets was evaluated using the Shapiro–Wilk test to determine whether parametric or nonparametric statistical methods were appropriate. Parametric analyses included one‐way analysis of variance with Tukey's post hoc test and unpaired *t*‐tests, whereas nonparametric analyses were performed using the Wilcoxon rank‐sum test. The significance threshold was set at *p* < 0.05. Correlation analyses were conducted using Pearson's correlation for parametric data and Spearman's correlation for nonparametric data.

## 3 RESULTS

### 3.1 Locomotor assessment

**FIGURE 2:**
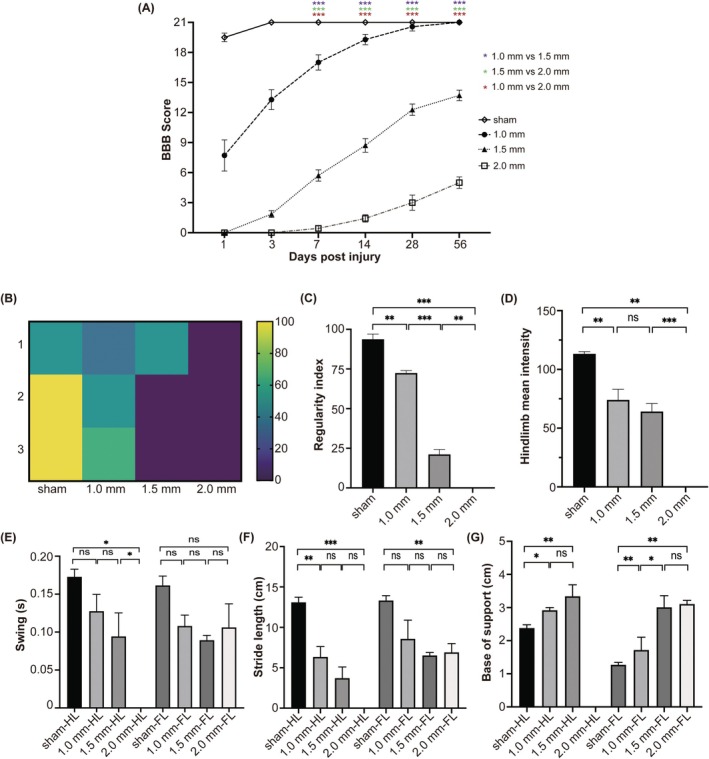
Locomotor assessment of the graded SCI (spinal cord injury) model. (A) Open‐field BBB (Basso, Beattie, Bresnahan) score at different time points postinjury. (B) Heat map illustrating the percentage of Ab regular step pattern across groups. The proportion of the Ab pattern decreases progressively with increasing injury severity. (C–G) Comparison of CatWalk gait analysis parameters among groups with different impact depths. *p < 0.05, **p < 0.01, ***p < 0.001; ns, non significant.

Gradually improved average and individual BBB scores (Figures 2A and S1F–H) were observed in all SCI groups over the 56‐day observation period. A significant difference (*p* < 0.001) in average BBB score was observed between the 1.0‐, 1.5‐, and 2.0‐mm groups at 7 dpi, and this disparity continued until the end of the observation period. Overall, the groups subjected to greater impact depths demonstrated lower average BBB scores at the corresponding time points than those subjected to lower impact depths.

The selected parameters of the CatWalk gait analysis revealed a gradient change pattern that correlated with the impact depth (Figure 2B–G). In the 2.0‐mm group, some hindlimb parameters, including mean intensity, swing duration, stride length, and base of support, could not be recorded because of the inability of the hind limbs to fully support the weight; consequently, these values were assigned as 0 for further analysis. The heat map (Figure 2B) illustrates that the percentage of the Ab pattern was the highest in the sham group and gradually decreased with increasing impact depth. Regarding regularity index (RI), significant differences were observed among the four groups (Figure 2C, *p* < 0.01), indicating varying degrees of impairment in interlimb coordination among the injury groups. Regarding hindlimb mean intensity, although no significant difference was observed between the 1.0‐ and 1.5‐mm groups, a decreasing trend was significant across all three injury groups (Figure 2D). Regarding swing duration, a graded decreasing pattern in the hind limbs was observed across the injury groups (Figure 2E). With increasing impact depth, the hindlimb stride length exhibited a decreasing trend (Figure 2F), whereas the hindlimb base of the support progressively increased (Figure 2G). These results collectively demonstrated a graded impairment in hindlimb function across the 1.0‐, 1.5‐, and 2.0‐mm groups.

### 3.2 MRI morphometry of the injured spinal cord

**FIGURE 3:**
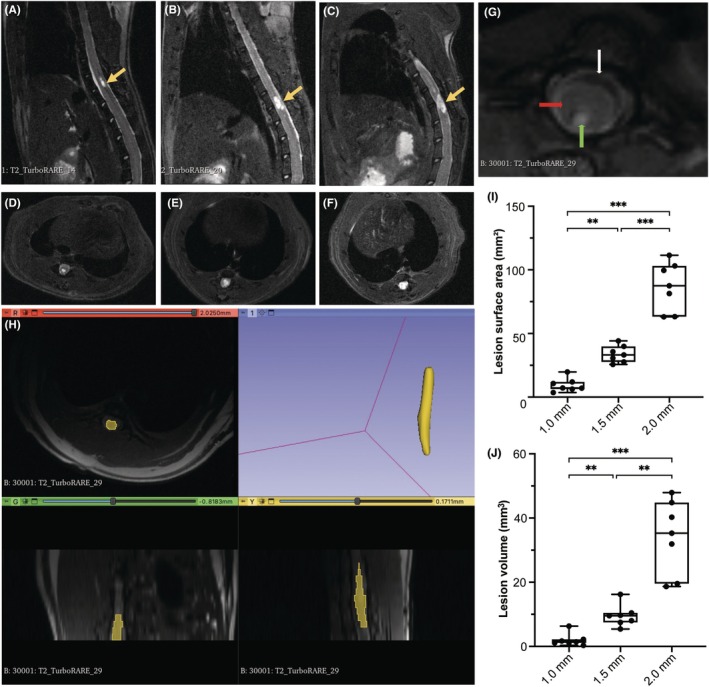
MRI (magnetic resonance imaging) acquisition and analysis of the lesion region. (A–F) Representative T2‐weighted magnetic resonance images of graded SCI (spinal cord injury) of rats. (A–C) Sagittal, (D–F) axial. Yellow arrow: the lesion region. (G) Representative T2‐weighted axial images of lesion region. Green arrow: cyst with fluid. Red arrow: surrounding scar tissue. White arrow: normal spinal cord tissue. (H) Segmentation and reconstruction of the lesion region. (I, J) Box‐and‐whisker plot of the surface area and volume of lesion region across different impact depths. **p < 0.01, ***p < 0.001.

The lesion regions across the three injury groups were assessed using sagittal and axial T2‐weighted images (Figure 3A–F). Axial T2‐weighted images showed lesion boundaries (Figure 3G), which were subsequently segmented for further analysis (Figure 3H). The lesion surface area progressively increased from 9.47 ± 2.01 mm^2^ in the 1.0‐mm group to 33.82 ± 2.49 mm^2^ in the 1.5‐mm group, and further to 87.05 ± 7.23 mm^2^ in the 2.0‐mm group. Similarly, the lesion volume increased from 2.04 ± 0.74 mm^3^ in the 1.0‐mm group to 9.60 ± 1.28 mm^3^ in the 1.5‐mm group and 34.36 ± 4.36 mm^3^ in the 2.0‐mm group. Statistical analysis revealed significant differences among the 1.0‐, 1.5‐, and 2.0‐mm injury groups in both lesion surface area (*p* < 0.01) and volume (*p* < 0.01) (Figure 3I,J). These findings indicate that the lesion size is positively correlated with increasing impact depth.

### 3.3 Histological assessment

**FIGURE 4:**
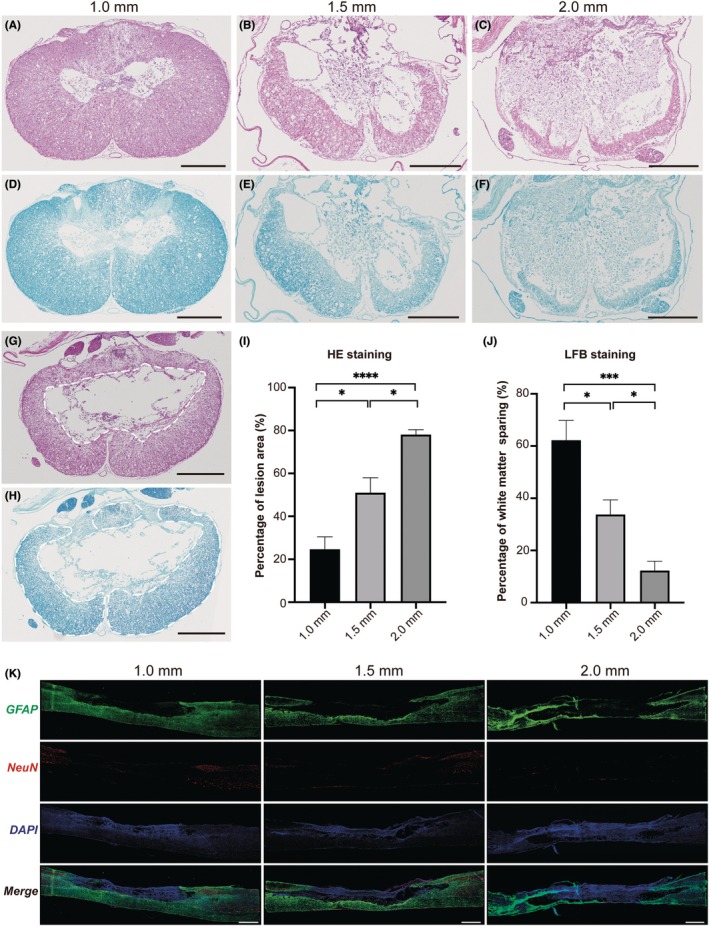
Histological assessment of the graded spinal cord injuries. (A–F) Representative images of HE (hematoxylin–eosin) and LFB (Luxol Fast Blue) staining of the injury epicenter. (G) Lesion area on HE staining. (H) White matter sparing on LFB staining. (I) Comparison of the percentage of lesion area on HE staining among three injury groups. (J) Comparison of the percentage of white matter sparing on LFB staining among three injury groups. (K) Representative images of GFAP (glial fibrillary acidic protein) and NeuN (neuronal nuclei) immunofluorescence staining at 56 dpi (day postinjury). Scale bar: A–H: 500 μm, K: 1 mm. *p < 0.05, ***p < 0.001, ****p < 0.0001.

Both HE and LFB staining of the lesion epicenter revealed significant disruption of spinal cord tissues (Figure 4A–F). The percentage of lesion area relative to the total spinal cord area was quantified via HE staining (Figure 4G). The percentage of white matter sparing relative to the total spinal cord was quantified using LFB staining (Figure 4H). The lesion percentage increased from 24.67% ± 5.77% in the 1.0‐mm group to 50.99% ± 6.97% in the 1.5‐mm group and 78.07% ± 2.32% in the 2.0‐mm group. The percentage of white matter sparing decreased from 62.25% ± 7.57% in the 1.0‐mm group to 33.78% ± 5.56% in the 1.5‐mm group and 12.26% ± 3.63% in the 2.0‐mm group. The results revealed a significant enlargement of the lesion area (Figure 4I, *p* < 0.05) and a reduction in the percentage of white matter sparing (Figure 4J, *p* < 0.05) with increasing impact depth. Immunofluorescence analysis of the sagittal sections demonstrated dynamic changes in response to increasing impact depth. The astrocytic response (GFAP) marked an expanding injured area (Figures 4K and S2A,B), which was accompanied by a progressive loss of neurons (NeuN) (Figure 4K). We also observed infiltration of microglia/macrophages (IBA1) at the lesion core and injury border (Figure S2A), alongside a reduction in oligodendrocytes (Olig2) adjacent to the injury (Figure S2B). These results delineated a graded tissue destruction across the three injury groups.

### 3.4 Correlation analysis

**FIGURE 5:**
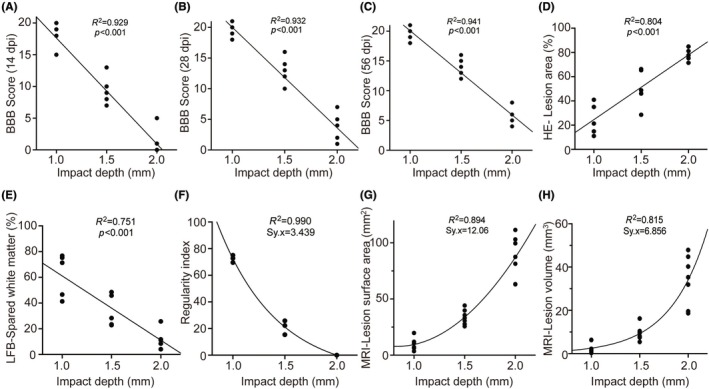
Correlation analysis of impact depth with different postinjury assessment parameters. (A–C) BBB (Basso, Beattie, Bresnahan) scores at 14, 28, and 56 dpi (day postinjury). (D) Percentage of lesion area on HE (hematoxylin–eosin) staining. (E) Percentage of white matter sparing on LFB (Luxol Fast Blue) staining. (F) Regularity index of CatWalk gait analysis. (G) Surface area of the lesion on MRI (magnetic resonance imaging). (H) Volume of the lesion on MRI.

Correlation analysis demonstrated significant associations between impact depth and various functional, histological, and imaging parameters, including BBB scores, percentage of histological lesion area, RI, MRI lesion surface area, and lesion volume (Figure 5). Particularly, impact depth exhibited a strong negative linear correlation with BBB scores on 14, 28, and 56 dpi (Figure 5A–C). Furthermore, impact depth positively correlated with the histological lesion area (Figure 5D) and negatively correlated with white matter sparing (Figure 5E). The RI showed a significant negative nonlinear correlation with impact depth (Figure 5F). Finally, MRI‐derived lesion surface area and volume demonstrated significant positive nonlinear correlations with impact depth (Figure 5G,H).

### 3.5 PCA of bulk RNA sequencing

**FIGURE 6:**
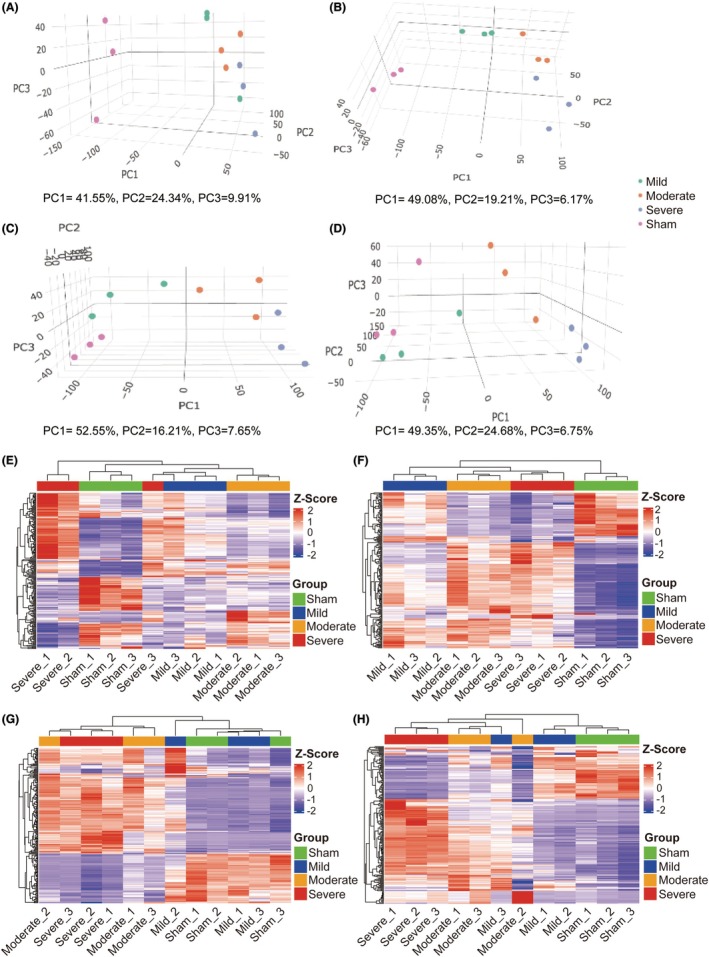
Hierarchical clustering of graded SCI (spinal cord injury) by transcriptome gene expression profiles. (A–D) PCA (principal component analysis) of the expressed transcripts with three principal components (PC1, 2, and 3) was performed to demonstrate the source of variance at (A) 1 dpi, (B) 3 dpi, (C) 14 dpi, and (D) 56 dpi. (E–H) Heat map clustering of gene expression profiles of graded SCI at (E) 1 dpi, (F) 3 dpi, (G) 14 dpi, and (H) 56 dpi.

To explore the variance in transcript expression among the different groups, PCA was conducted at multiple postinjury time points using two (Figure S3) and three principal components (Figure 6A–D). As illustrated in Figure 6A, the PC score plots show a distribution of variance in the expressed transcripts at 1 dpi. The contributions of PC1, PC2, and PC3 were 41.55%, 24.34%, and 9.91%, respectively. The three biological replicates of the sham group clustered together, whereas the replicates of the three injury groups clustered together, indicating that the transcriptome profile of the injury group differed significantly from that of the sham group. At 3 dpi (Figure 6B), the three biological replicates were closely clustered within each group, whereas a distinct separation was observed between the different injury groups, with all the injury groups positioned distinctly from the sham group. Similar distribution patterns were observed at 14 (Figure 6C) and 56 dpi (Figure 6D). These findings revealed substantial transcriptomic variance among groups with different injury severities.

### 3.6 Hierarchical clustering analysis of gene expression with varying injury severities

To further identify the transcriptome discrepancies among groups and similarities within each group, the top 200 genes with the highest variance in the expression matrix were selected for hierarchical clustering (Figure 6E–H). The clustering results revealed that, in most cases, biological replicates within the same injury and sham groups were grouped together at each time point postinjury, indicating high intragroup similarity. This transcriptome pattern further confirmed the graded injury in each group.

### 3.7 GSEA and GO enrichment of DEGs

GSEA was used to assess the presence of general biological processes within each injury group (Figure S4). At 1 dpi (Figure S4A), 3 dpi (Figure S4B), and 14 dpi (Figure S4C), all selected biological processes were enriched in the three injury groups. At 56 dpi (Figure S4D), although the GO term “axon ensheathment in central nervous system” was not significantly enriched in the mild group (*p* > 0.05), all the selected biological processes were enriched in the moderate and severe groups. For GO enrichment (Figure S5), the results demonstrated significant differences among the top 15 enriched biological processes in each group at 1 dpi (Figure S5A), 3 dpi (Figure S5B), 14 dpi (Figure S5C), and 56 dpi (Figure S5D).

### 3.8 Gene expression trend analysis and identification of key biological pathways associated with injury severity

**FIGURE 7:**
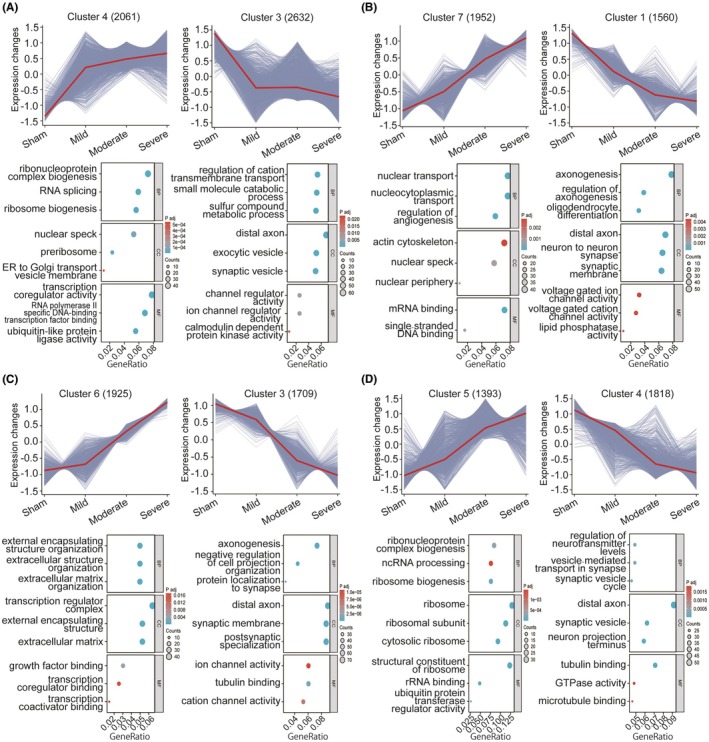
Gene clusters exhibiting consistent upregulation or downregulation trends and their top three enriched GO (Gene Ontology) terms at (A) 1 dpi (day postinjury), (B) 3 dpi, (C) 14 dpi, and (D) 56 dpi.

The clustering results for each time point are shown in Figure S6. Representative clusters exhibiting a consistent upregulation or downregulation trend in expression relative to injury severity were selected for further analysis; GO enrichment analysis was performed on the genes within these clusters. At 1 dpi, cluster 4 containing 2061 genes exhibited a consistent upregulation trend (Figure 7A). GO enrichment of these genes revealed that “ribonucleoprotein complex biogenesis,” “RNA splicing,” and “ribosome biogenesis” were the top three enriched biological processes; “nuclear speck,” “preribosome,” and “ER to Golgi transport vesicle membrane” were the top three cellular components; and “transcription coregulator activity,” “RNA polymerase II–specific DNA‐binding transcription factor binding,” and “ubiquitin‐like protein ligase activity” were the top three molecular functions. Cluster 3, consisting of 2632 genes, exhibited a consistent downregulation trend (Figure 7A). GO enrichment of these genes revealed that “regulation of cation transmembrane transport,” “small molecule catabolic process,” and “sulfur compound metabolic process” were the top three enriched biological processes; “distal axon,” “exocytic vesicle,” and “synaptic vesicle” were the top three cellular components; “channel regulator activity,” “ion channel regulator activity,” and “calmodulin‐dependent protein kinase activity” were the top three enriched molecular functions. GO enrichments for the consistently upregulated and downregulated clusters at other time points are shown in Figure 7B (3 dpi), Figure 7C (14 dpi), and Figure 7D (56 dpi).

## 4 DISCUSSION

Consistent, reproducible, and quantifiable SCI models facilitate the investigation of underlying pathophysiological mechanisms and the development of effective therapeutic interventions for clinical applications. In this study, we developed a graded SCI model by directly configuring the impact depth, which induced different levels of stress and strain within the spinal cord tissue.^22^ Because impact velocity can interact with impact depth to affect the severity of contusion injury,^23^ we fixed it at a relatively high speed (3 m/s) to better replicate clinical scenarios. The dwell time was set at 5 s because a longer dwell time would induce compression injury. Previous studies showed that the addition of a compression component to a contusion injury exacerbates tissue damage and impairs functional recovery.^24^, ^25^ These findings align with evidence indicating that compression after contusion SCI alters perfusion, disrupts energy metabolism, exacerbates hemorrhage, and affects revascularization.^26^, ^27^ Therefore, our dual‐component injury model enhances our understanding of the biomechanical contributors to secondary injury and highlights the importance of considering primary injury biomechanics in the development and application of SCI therapies. Another characteristic of patients with clinical SCI is an increasing proportion of incomplete injuries, which underlies varying degrees of spontaneous recovery. Natural recovery can influence acute management decisions, establish functional goals, and facilitate individualized rehabilitation strategies. Thus, modeling incomplete injuries of graded severity is essential for the development of individualized therapeutic approaches in clinical settings. Previous studies have identified tissue displacement as a critical determinant of injury severity in contusion SCI models.^5^, ^28^ In our study, we further demonstrated that precise control of impact depths of 1.0, 1.5, and 2.0 mm, with impact velocity at 3 m/s and dwell time at 5 s, can induce graded injuries on adult rats. Because spontaneous recovery in rats typically reaches a plateau at 6–8 weeks after injury,^29^ we set our final observation time point at 56 dpi to capture the entire recovery trajectory. A comprehensive evaluation and correlation analysis validated that there are significant associations between impact depth and various functional, histological, and imaging measures. These results are consistent with previous studies.^6^, ^7^

Our study further revealed transcriptomic changes after graded SCI. The PCA results demonstrated distinct clustering of each group, with a similar distribution of biological replicates in each group. Heat map–based hierarchical clustering further indicated that although some samples clustered near adjacent groups, the majority of biological replicates could be clustered together at different time points postinjury. These clustering analyses indicated that injury severity significantly affected transcriptional profiles over time. The clear separation of groups based on gene expression suggests that transcriptomic changes can be used to distinguish different injury severities. Furthermore, GSEA and GO analyses revealed that although all injury groups shared core SCI‐related biological responses, the general pattern of biological processes activated postinjury differed among the injury groups.

Additionally, we identified potential pathways that consistently underwent upregulation or downregulation after SCI by combining gene expression trend analysis. At 1 dpi, the top three enriched biological processes of the consistently upregulated genes correlated with RNA metabolism, including transcription, processing, and translation. The top three cellular component terms indicated predominantly nuclear localization (involving RNA splicing and ribosome assembly), while contributing to protein transport and cellular trafficking. Regarding molecular function, the top elements highlighted transcriptional regulation via interactions between transcription factors and RNA polymerase II. This enrichment pattern coincides with pathophysiological events in the acute phase of SCI, such as the endoplasmic reticulum stress response and inflammation.^30^, ^31^, ^32^, ^33^ The top three enriched biological processes of consistently downregulated genes indicated the suppression of ion transport across membranes and reduced sulfur metabolism. The top cellular component terms suggested impairment in axonal transport, synaptic maintenance, or neuronal communication. The top molecular function terms were the dysregulation of ion channels, suppression of ion channel regulators, and reduction in calmodulin‐dependent kinase activity. These enriched GO terms were aligned with pathological mechanisms of neuroinflammation and axonal dysfunction in the acute phase of SCI.^34^, ^35^, ^36^ Using a similar analytical framework, we examined the biological events that were continuously activated or inhibited at 3, 14, and 56 dpi. These biological events may serve as potential directions for screening biomarkers to assess injury severity in future studies.

Despite the contributions of this study, its limitations remain unaddressed. First, the 68099 II Precise Impactor used in our experiments has a maximum dwell time of 5 s, which may not be sufficient to model the prolonged compression to better mimicking the biomechanical features of clinical SCI. Second, unlike hard clustering methods, *Mfuzz* used soft clustering, allowing each gene to be assigned membership to multiple clusters rather than being strictly categorized into a single cluster.^21^ This inherent flexibility introduces variability within clusters, where some genes may not perfectly conform to the dominant trend yet still exhibit partial similarity. Third, bulk RNA sequencing provides only averaged gene expression profiles across heterogeneous cell populations, which may mask cell‐type‐specific transcriptional changes that are critical for understanding the underlying mechanisms of SCI. In contrast, single‐cell RNA sequencing allows the identification of cell‐type‐specific transcriptional profiles and intercellular communication, which may reveal in‐depth pathophysiological processes such as autophagy,^37^ blood–spinal cord barrier reconstruction,^38^ and dynamic conversions of microglia.^39^

In conclusion, we established a graded SCI model by configuring the impact depth as a key determinant, which was subsequently validated through a comprehensive assessment. As the impact depth increased, the injured rats exhibited worse hindlimb function, larger imaging lesion regions, more severe histological destruction, and distinct transcriptomic profiles. Furthermore, using this graded injury model, we offered insights into injury severity–dependent transcriptomic changes and identified the biological mechanisms associated with injury severity. Future research should focus on these severity‐related mechanisms to identify potential biomarkers for injury classification and to enhance clinical management strategies for SCI.

## AUTHOR CONTRIBUTIONS

**Gang Zhou:** Conceptualization; formal analysis; investigation; methodology; visualization; writing – original draft. **Yue Hu:** Data curation; methodology; software. **Zhimin Li:** Methodology; visualization. **Shiyuan Han:** Software; visualization. **Yuejia Li:** Data curation; validation. **Yongning Li:** Conceptualization; supervision; writing – review and editing. **Jun Gao:** Conceptualization; funding acquisition; resources; supervision; writing – review and editing.

## FUNDING INFORMATION

This work was supported by the Yinhua Public Welfare Foundation.

## CONFLICT OF INTEREST STATEMENT

The authors declare no conflicts of interest.

## ETHICS STATEMENT

The research protocol involved in this article has been approved by Animal Ethics Committee of Peking Union Medical College Hospital (Approval Number: XHDW‐2024‐74). All the experiments were performed in accordance with guidelines of Animal Experiment Center of Peking Union Medical College Hospital and the National Institutes of Health (NIH) Guide for the Care and Use of Laboratory Animals.

## Supporting information


**Figure S1.** Method for establishing the SCI (spinal cord injury) model and BBB (Basso, Beattie, Bresnahan) scores of each individual rat. (A) The 68099 II Precise Impactor (RWD). (B) Immobilization, position adjustment, and zero‐point calibration of the rat on the impactor system. (C) Schematic diagram to illustrate the impact depth. (D, E) Intraoperative images of spinal cord before and after injury, respectively. An obvious subdural hematoma is observed at the injury site. (F–H) Line graph of BBB scores of each individual rat in the (F) 1.0‐, (G) 1.5‐, and (H) 2.0‐mm groups.

**Figure S2.** Immunofluorescence staining of the spinal cord injury epicenter at 56 dpi (day postinjury). (A) Representative images of double labeling for glial fibrillary acidic protein (GFAP) and ionized calcium‐binding adapter molecule 1 (IBA1). (B) Representative images of double labeling for GFAP and oligodendrocyte transcription factor 2 (Olig2).

**Figure S3.** Principal component analysis (PCA) of the expressed transcripts with two principal components at (A) 1 day postinjury (dpi), (B) 3 dpi, (C) 14 dpi, and (D) 56 dpi.

**Figure S4.** Ridge plots of gene set enrichment analysis (GSEA) for injured spinal cord transcriptome at (A) 1 day postinjury (dpi), (B) 3 dpi, (C) 14 dpi, and (D) 56 dpi.

**Figure S5.** Gene Ontology (GO) enrichment analysis for injured spinal cord transcriptome at (A) 1 day postinjury (dpi), (B) 3 dpi, (C) 14 dpi, and (D) 56 dpi.

**Figure S6.** Gene expression trend analysis for injured spinal cord transcriptome at (A) 1 day postinjury (dpi), (B) 3 dpi, (C) 14 dpi, and (D) 56 dpi.

**Table S1.** Description of the selected CatWalk XT parameters.
**Table S2.** Summary of sequence assembly and mapping statistics.

Data S1.

## Data Availability

The raw sequencing data generated in this study have been deposited in the NCBI Sequence Read Archive (SRA) under BioProject accession PRJNA1249449. Additional datasets analyzed in the study are available on request from the corresponding author.
