# Supplementary figures and images for: Establishment and multifaceted characterization of a graded spinal cord injury model based on graduated impact depth
Source: Animal Model Exp Med. 2026 Feb 27;9(4):809–21. doi: 10.1002/ame2.70150 (PMC13242734; doi:10.1002/ame2.70150)

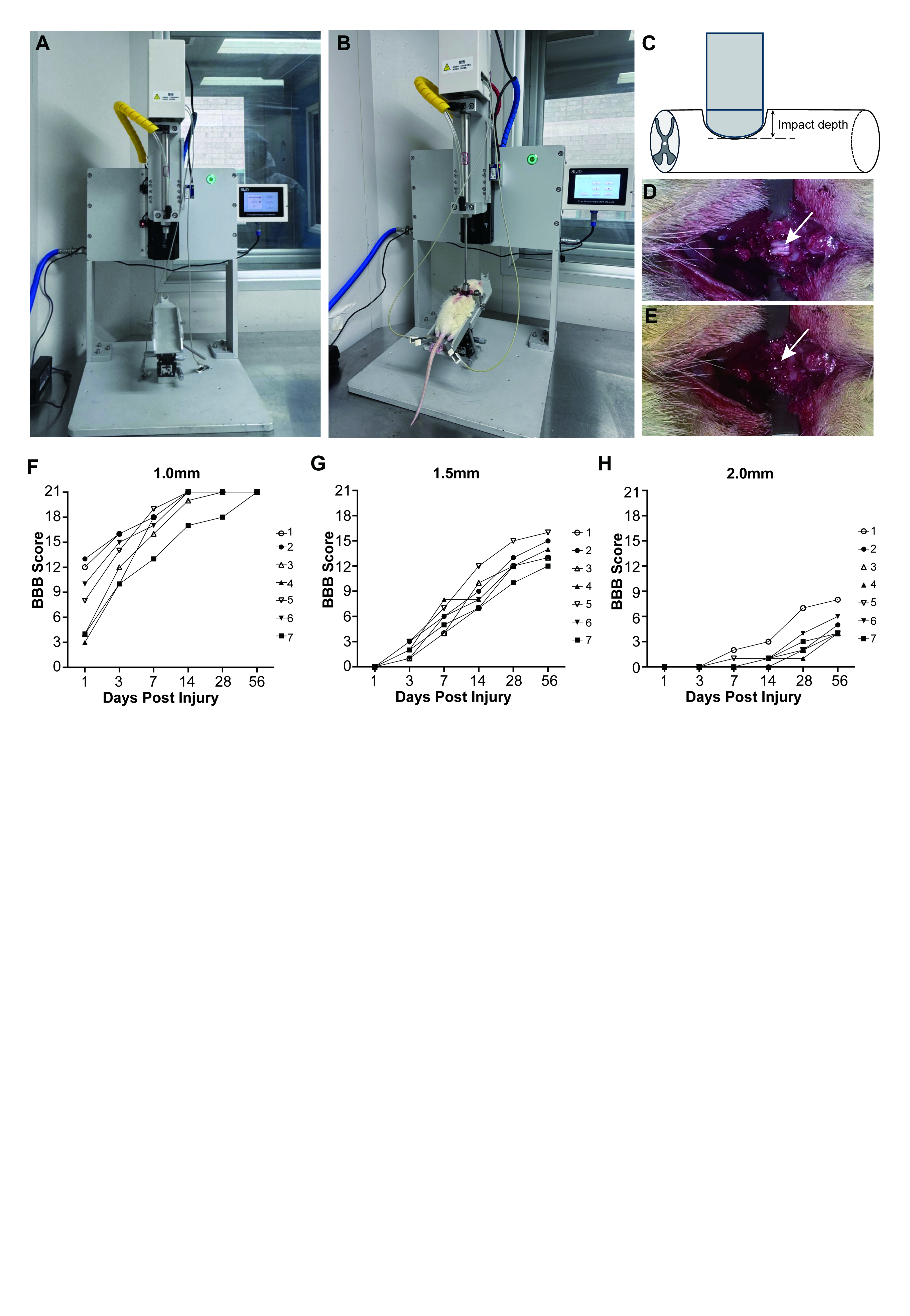

Supplement: Supplementary file 1 — Figure S1. Method for establishing the SCI (spinal cord injury) model and BBB (Basso, Beattie, Bresnahan) scores of each individual rat. (A) The 68099 II Precise Impactor (RWD). (B) Immobilization, position adjustment, and zero‐point calibration of the rat on the impactor system. (C) Schematic diagram to illustrate the impact depth. (D, E) Intraoperative images of spinal cord before and after injury, respectively. An obvious subdural hematoma is observed at the injury site. (F–H) Line graph of BBB scores of each individual rat in the (F) 1.0‐, (G) 1.5‐, and (H) 2.0‐mm groups. [file AME2-9-809-s005.tif]

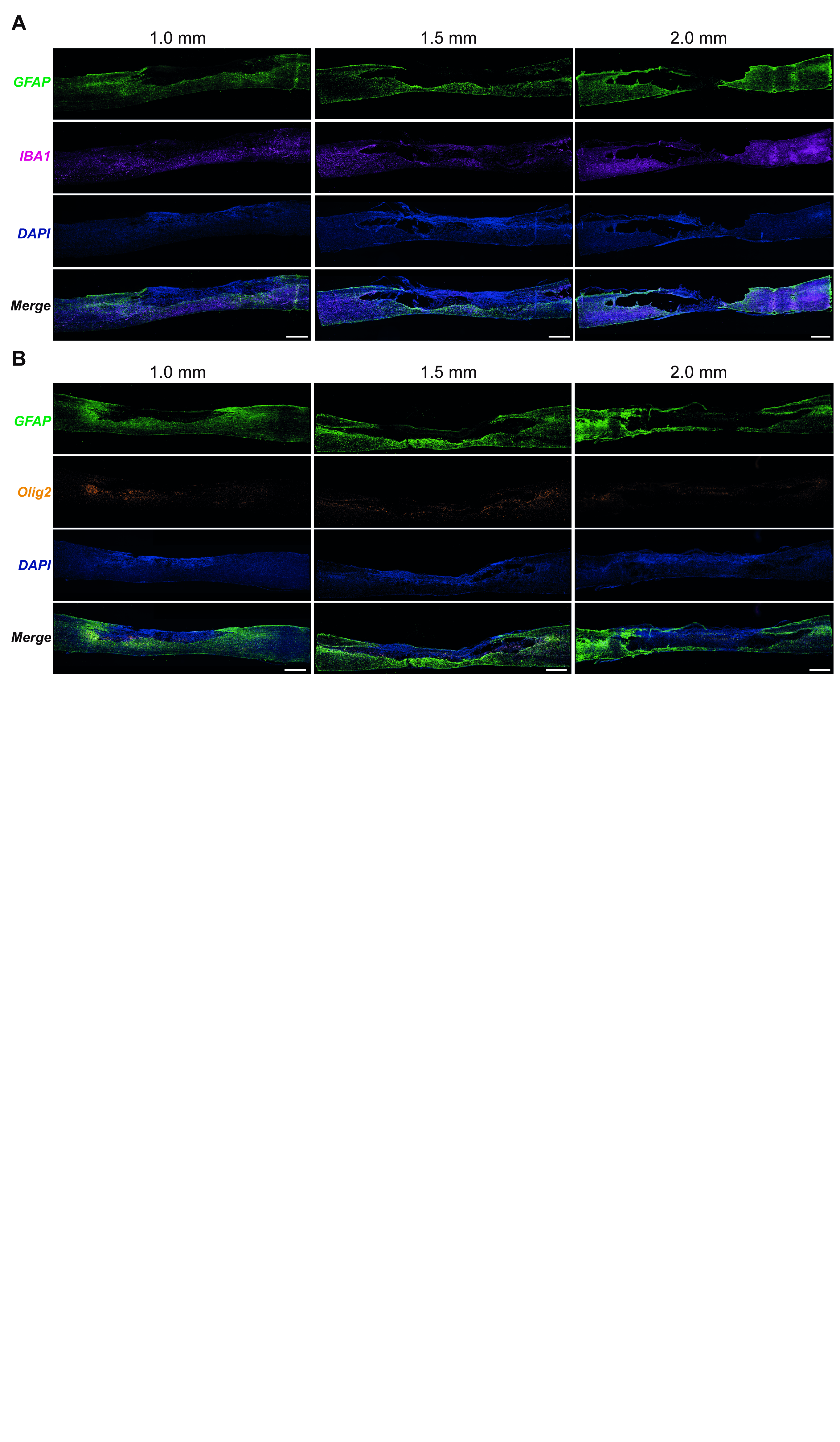

Supplement: Supplementary file 2 — Figure S2. Immunofluorescence staining of the spinal cord injury epicenter at 56 dpi (day postinjury). (A) Representative images of double labeling for glial fibrillary acidic protein (GFAP) and ionized calcium‐binding adapter molecule 1 (IBA1). (B) Representative images of double labeling for GFAP and oligodendrocyte transcription factor 2 (Olig2). [file AME2-9-809-s006.tif]

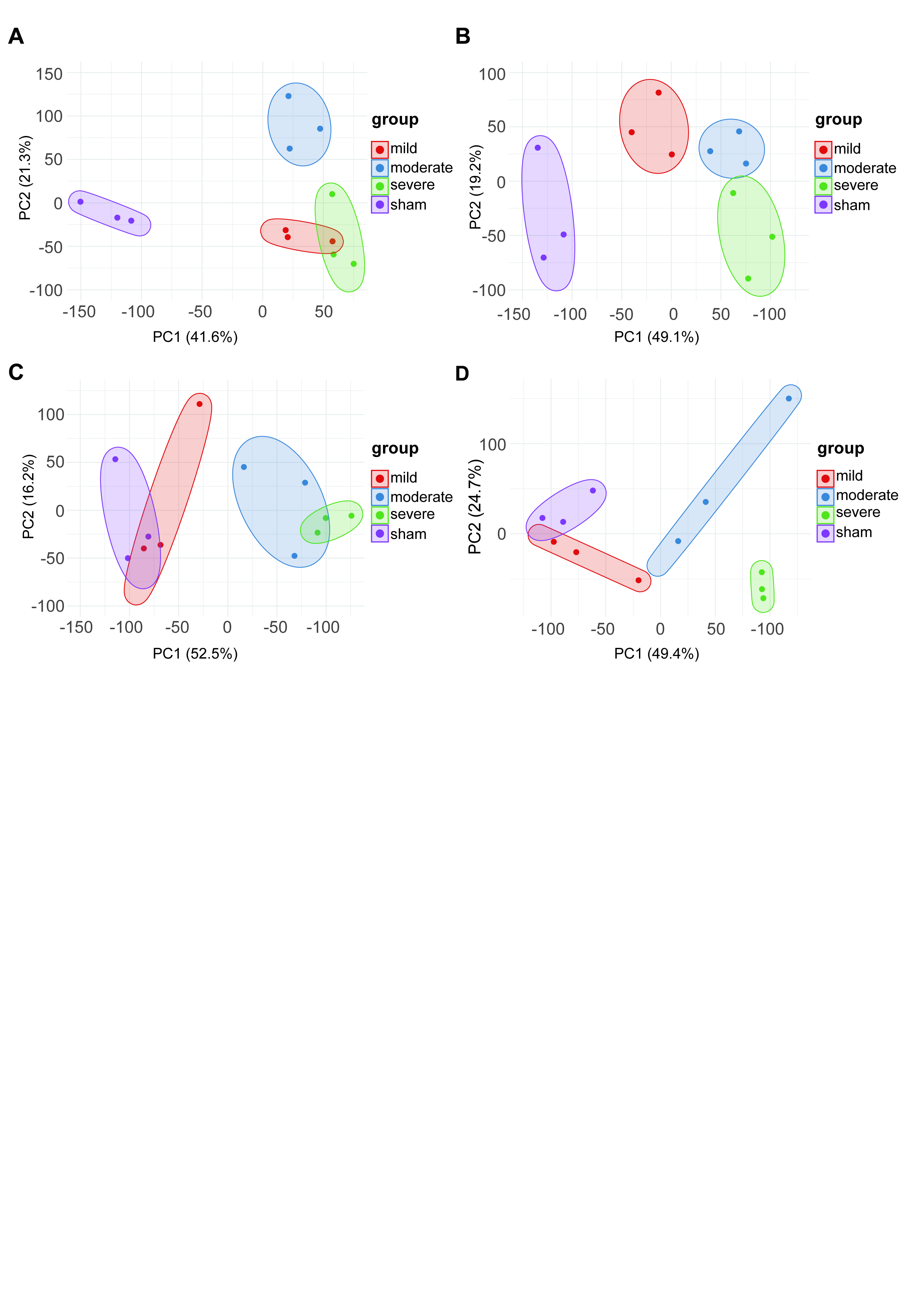

Supplement: Supplementary file 3 — Figure S3. Principal component analysis (PCA) of the expressed transcripts with two principal components at (A) 1 day postinjury (dpi), (B) 3 dpi, (C) 14 dpi, and (D) 56 dpi. [file AME2-9-809-s003.tif]

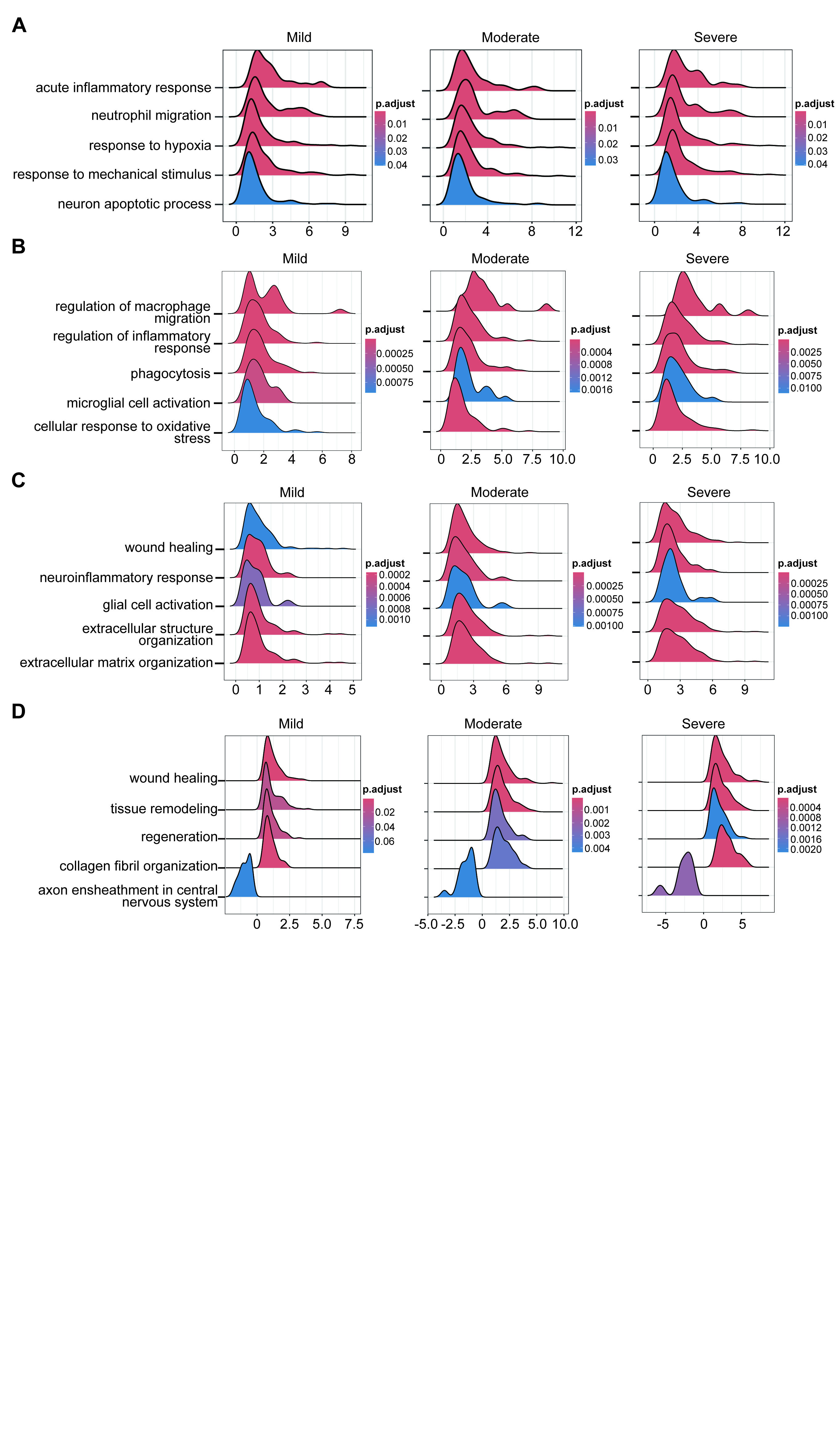

Supplement: Supplementary file 4 — Figure S4. Ridge plots of gene set enrichment analysis (GSEA) for injured spinal cord transcriptome at (A) 1 day postinjury (dpi), (B) 3 dpi, (C) 14 dpi, and (D) 56 dpi. [file AME2-9-809-s004.tif]

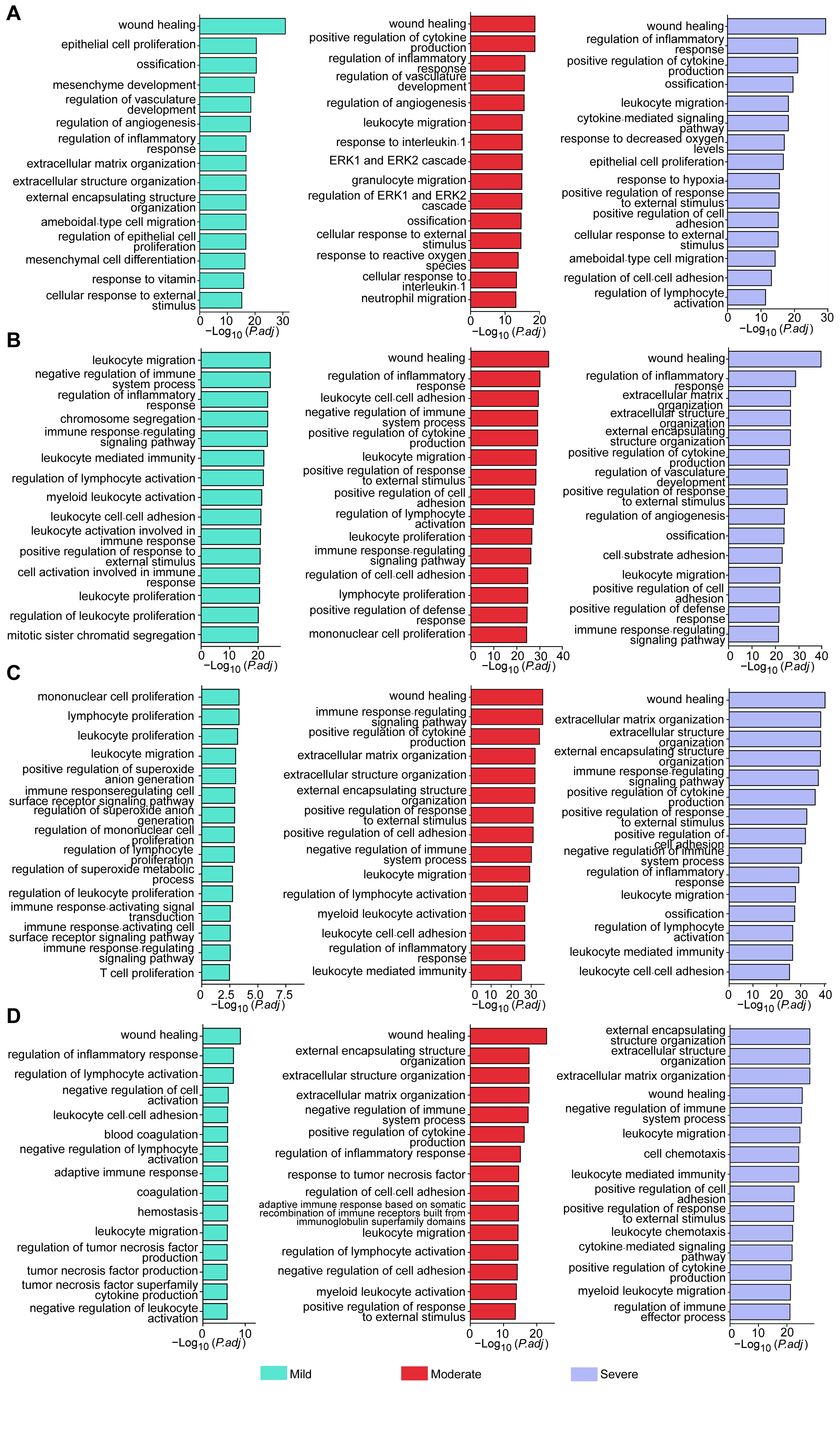

Supplement: Supplementary file 5 — Figure S5. Gene Ontology (GO) enrichment analysis for injured spinal cord transcriptome at (A) 1 day postinjury (dpi), (B) 3 dpi, (C) 14 dpi, and (D) 56 dpi. [file AME2-9-809-s001.tif]

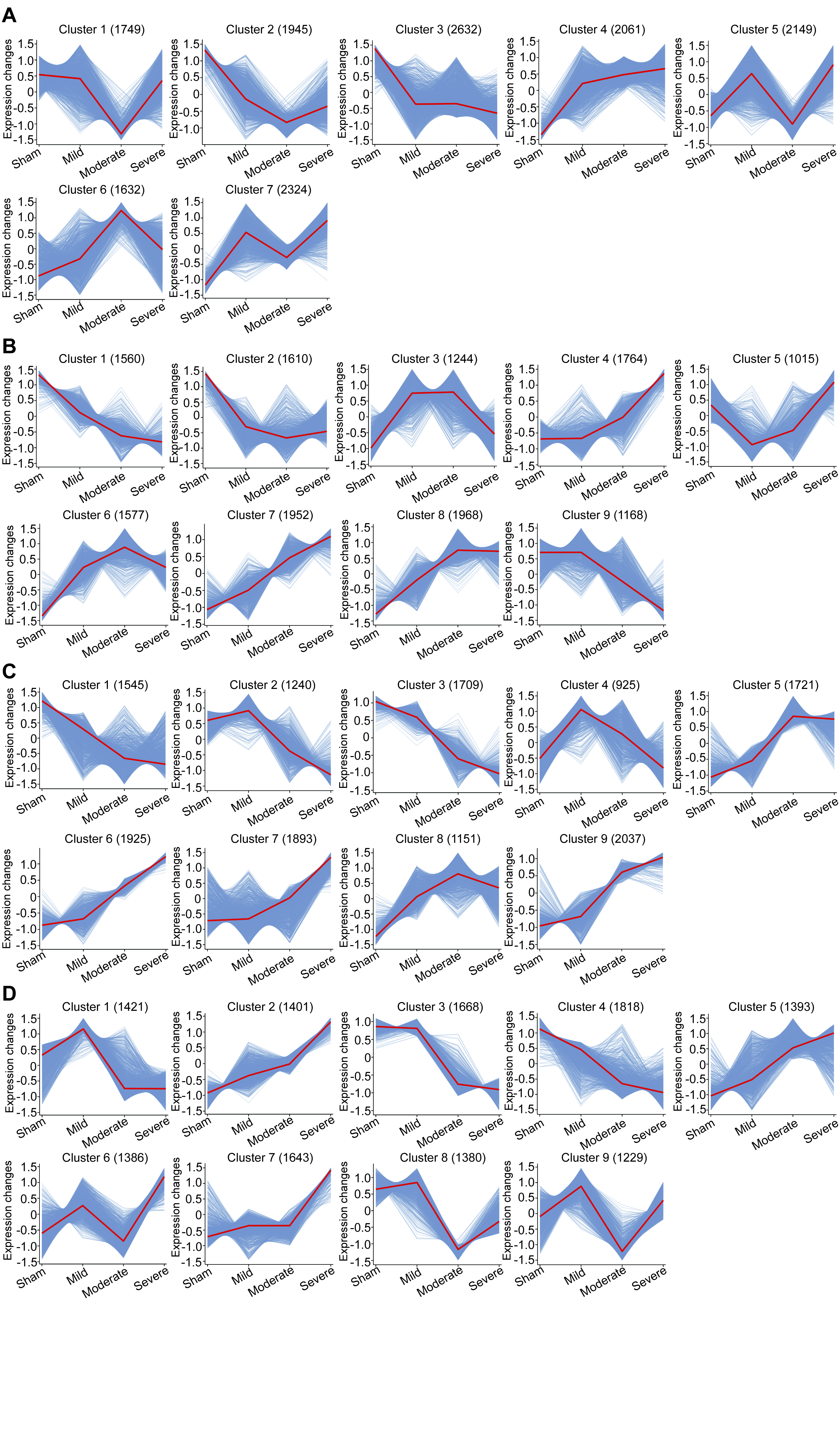

Supplement: Supplementary file 6 — Figure S6. Gene expression trend analysis for injured spinal cord transcriptome at (A) 1 day postinjury (dpi), (B) 3 dpi, (C) 14 dpi, and (D) 56 dpi. [file AME2-9-809-s002.tif]
