# Supplementary material for: Establishment and multifaceted characterization of a graded spinal cord injury model based on graduated impact depth
Source: Animal Model Exp Med. 2026 Feb 27;9(4):809–21. doi: 10.1002/ame2.70150 (PMC13242734; doi:10.1002/ame2.70150)
Supplement: Supplementary file 7 — Table S1. Description of the selected CatWalk XT parameters. Table S2. Summary of sequence assembly and mapping statistics. [file AME2-9-809-s007.docx]

**Supplementary Table 1**. Description of the selected CatWalk XT^®^ parameters

| Parameters | Description |
| --- | --- |
| Ab pattern percentage | Describe the prevalence of Ab regular step pattern, which is the most preferred pattern in intact rat. ﻿The value decreases and recovers only a little following contusion injuries. |
| ﻿Regularity index | Quantify the interlimb coordination by measuring the proportion of steps that conform to a normal gait pattern relative to the total number of paw placements. A reduced RI indicates impaired coordination between limbs. |
| Hindlimb mean intensity | Describe the pressure applied by the hind paws during movement, indicating the extent of weight-bearing capability. |
| Swing duration | Describe ﻿the interval during which the foot does not contact the ground, which is significantly shortened in both forelimbs and hindlimbs following contusion injuries . |
| Stride length | Describe the distance between two consecutive steps of the same paw, which tends to shorten in both forelimbs and hindlimbs following contusive injuries . |
| Base of support | ﻿Describe the mean width between the hind paws, which tends to increase following contusion injuries . |

**Supplementary Table 2.** Summary of ﻿sequence assembly and mapping statistics

| **﻿Sample name** | **﻿Number of Raw reads** | **﻿Number of Clean reads** | **Average read length (bp)** | **﻿Clean bases**  **(bp)** | **﻿Q20 (%)** | **﻿Q30 (%)** | **﻿GC (%)** | **﻿Total mapped** | **﻿Multiple mapped** | **﻿Uniquely mapped** |
| --- | --- | --- | --- | --- | --- | --- | --- | --- | --- | --- |
| ﻿Sham-1 | ﻿42212232 | ﻿41802756 | ﻿145.31 | ﻿6074264750 | ﻿98.06 | 94.47 | 47.33 | ﻿40288349  (96.3773%) | 3010396  (7.20143%) | 37277953  (89.1758%) |
| Sham-2 | ﻿41594992 | ﻿41286976 | ﻿145.42 | ﻿6003893112 | ﻿97.76 | 93.65 | 47.52 | ﻿39693057  (96.1394%) | 2631042  (6.37257%) | 37062015  (89.7668%) |
| Sham-2 | ﻿37379670 | ﻿37152894 | ﻿146.66 | ﻿5448765312 | ﻿97.79 | 93.71 | 47.43 | ﻿35727583  (96.1637%) | 2291593  (6.16801%) | 33435990  (89.9957%) |
| ﻿SCI-D1-mild-1 | ﻿44069506 | ﻿43777168 | ﻿147.13 | ﻿6440941976 | ﻿98.40 | 95.13 | 47.29 | ﻿42333278  (96.7017%) | 2832550  (6.47038%) | 39500728  (90.2313%) |
| ﻿SCI-D1-mild-2 | ﻿40386874 | ﻿40122462 | ﻿146.68 | ﻿5885160376 | ﻿98.39 | 95.07 | 46.96 | ﻿38818772  (96.7507%) | 2685112  (6.69229%) | 36133660  (90.0584%) |
| ﻿SCI-D1-mild-3 | ﻿50166818 | ﻿49829552 | ﻿147.11 | ﻿7330307582 | ﻿98.34 | 94.99 | 47.29 | ﻿48161741  (96.653%) | 3314001  (6.65067%) | 44847740  (90.0023%) |
| ﻿SCI-D1-moderate-1 | ﻿49763422 | ﻿49411892 | ﻿146.62 | ﻿7244977652 | ﻿98.40 | 95.15 | 46.98 | ﻿47735173  (96.6066%) | 3328459  (6.73615%) | 44406714  (89.8705%) |
| ﻿SCI-D1-moderate-2 | ﻿48977716 | ﻿48679648 | ﻿146.90 | ﻿7151250211 | ﻿98.37 | 95.06 | 47.10 | ﻿47094408  (96.7435%) | 3213878  (6.6021%) | 43880530  (90.1414%) |
| ﻿SCI-D1-moderate-3 | ﻿44670552 | ﻿44383572 | ﻿146.58 | ﻿6505830789 | ﻿98.36 | 95.04 | 47.13 | ﻿42882795  (96.6186%) | 2854640  (6.43175%) | 40028155  (90.1869%) |
| ﻿SCI-D1-severe-1 | ﻿45803034 | ﻿45498562 | ﻿146.82 | ﻿6680262380 | ﻿98.35 | 95.01 | 47.33 | ﻿44010636  (96.7297%) | 3231831  (7.10315%) | 40778805  (89.6266%) |
| ﻿SCI-D1-severe-2 | ﻿44796080 | ﻿44478326 | ﻿146.84 | ﻿6531207152 | ﻿98.40 | 95.14 | 47.31 | ﻿42998651  (96.6733%) | 3175781  (7.14006%) | 39822870  (89.5332%) |
| ﻿SCI-D1-severe-3 | ﻿45537734 | ﻿45221532 | ﻿147.09 | ﻿6651475080 | ﻿98.34 | 94.98 | 47.27 | ﻿43712743  (96.6636%) | 3020271  (6.67883%) | 40692472  (89.9847%) |
| ﻿SCI-D3-mild-1 | ﻿43307898 | ﻿43002588 | ﻿ ﻿145.78 | ﻿6269059839 | ﻿98.10 | 94.52 | 47.46 | ﻿41530382  (96.5765%) | 3086672  (7.17787%) | 38443710  (89.3986%) |
| ﻿SCI-D3-mild-2 | ﻿44525014 | ﻿44196782 | ﻿ ﻿145.28 | ﻿6420752069 | ﻿97.94 | 94.07 | 47.60 | ﻿2667543  (96.5399%) | 3043497  (6.88624%) | 39624046  (89.6537%) |
| ﻿SCI-D3-mild-3 | ﻿45215756 | ﻿44900564 | ﻿ ﻿145.86 | ﻿6549101190 | ﻿97.79 | 93.70 | 47.81 | ﻿43272965  (96.3751%) | 3062633  (6.82092%) | 40210332  (89.5542%) |
| ﻿SCI-D3-moderate-1 | ﻿43233710 | ﻿42999728 | ﻿146.22 | ﻿6287239291 | ﻿98.14 | 94.63 | 47.89 | ﻿41569349  (96.6735%) | 3163384  (7.35675%) | 38405965  (89.3168%) |
| ﻿SCI-D3-moderate-2 | ﻿43020126 | ﻿42760572 | ﻿146.37 | ﻿6258872559 | ﻿98.00 | 94.29 | 47.87 | ﻿41244122  (96.4536%) | 3028255  (7.08189%) | 38215867  (89.3717%) |
| ﻿SCI-D3-moderate-3 | ﻿41438438 | ﻿41226206 | ﻿146.54 | ﻿6041439743 | ﻿97.82 | 93.74 | 48.04 | ﻿39845130  (96.65%) | 2943053  (7.13879%) | 36902077  (89.5112%) |
| ﻿SCI-D3-severe-1 | ﻿43758810 | ﻿43554344 | ﻿146.15 | ﻿6365562353 | ﻿98.03 | 94.32 | 48.18 | ﻿42133580  (96.738%) | 3347338  (7.68543%) | 38786242  (89.0525%) |
| ﻿SCI-D3-severe-2 | ﻿36779234 | ﻿36544956 | ﻿145.97 | ﻿5334295155 | ﻿97.88 | 93.88 | 48.00 | ﻿35278239  (96.5338%) | 2612226  (7.14798%) | 32666013  (89.3858%) |
| ﻿SCI-D3-severe-3 | ﻿42817588 | ﻿42501996 | ﻿145.76 | ﻿6195139253 | ﻿98.11 | 94.50 | 48.32 | ﻿41101918  (96.7059%) | 3273849  (7.70281%) | 37828069  (89.003%) |
| ﻿SCI-D14-mild-1 | ﻿42057744 | ﻿41749584 | ﻿145.71 | ﻿﻿6083289026 | ﻿97.92 | 94.00 | 47.38 | ﻿40248149  (96.4037%) | 2646838  (6.33979%) | 37601311  (90.0639%) |
| ﻿SCI-D14-mild-2 | ﻿42220464 | ﻿41905586 | ﻿145.24 | ﻿6086523178 | ﻿98.01 | 94.36 | 47.87 | ﻿40367599  (96.3299%) | 3297924  (7.86989%) | 37069675  (88.46%) |
| ﻿SCI-D14-mild-3 | ﻿43213768 | ﻿42815364 | ﻿146.09 | ﻿6254820887 | ﻿98.15 | 94.64 | 47.61 | ﻿ 41293391  (96.4453%) | 2628350  (6.1388%) | 38665041  (90.3065%) |
| ﻿SCI-D14-moderate-1 | ﻿43358754 | ﻿43062170 | ﻿146.29 | ﻿6299392328 | ﻿98.02 | 94.32 | 48.09 | ﻿41515095  (96.4073%) | 2915085  (6.76948%) | 38600010  (89.6379%) |
| ﻿SCI-D14-moderate-2 | ﻿42891958 | ﻿42628310 | ﻿146.31 | ﻿6237095288 | ﻿98.14 | 94.60 | 48.11 | ﻿41163914  (96.5647%) | 3092633  (7.25488%) | 38071281  (89.3099%) |
| ﻿SCI-D14-moderate-3 | ﻿44967234 | ﻿44781172 | ﻿146.32 | ﻿6552582823 | ﻿97.62 | 93.37 | 48.17 | ﻿43048811  (96.1315%) | 2893856  (6.46222%) | 40154955  (89.6693%) |
| ﻿SCI-D14-severe-1 | ﻿37108068 | ﻿36857548 | ﻿146.59 | ﻿5402980088 | ﻿97.61 | 93.26 | 48.26 | ﻿35460786  (96.2104%) | 2521669  (6.84166%) | 32939117  (89.3687%) |
| ﻿SCI-D14-severe-2 | ﻿36750614 | ﻿36527974 | ﻿146.69 | ﻿5358396936 | ﻿97.81 | 93.79 | 48.52 | ﻿35167835  (96.2764%) | 2539204  (6.9514%) | 32628631  (89.325%) |
| ﻿SCI-D14-severe-3 | ﻿38060174 | ﻿37749690 | ﻿145.46 | ﻿5491233199 | ﻿97.78 | 93.68 | 48.39 | ﻿36356962  (96.3106%) | 2552031  (6.7604%) | 33804931  (89.5502%) |
| ﻿SCI-D56-mild-1 | ﻿39106772 | ﻿38932200 | ﻿146.17 | ﻿5690863908 | ﻿96.94 | 91.88 | 47.12 | ﻿37283822  (95.766%) | 2562962  (6.58314%) | 34720860  (89.1829%) |
| ﻿SCI-D56-mild-2 | ﻿49480222 | ﻿49228614 | ﻿146.30 | ﻿7202065672 | ﻿96.78 | 91.55 | 47.20 | ﻿47068609  (95.6123%) | 3168164  (6.43561%) | 43900445  (89.1767%) |
| ﻿SCI-D56-mild-3 | ﻿40885774 | ﻿40583482 | ﻿145.40 | ﻿5900730879 | ﻿96.84 | 91.65 | 46.88 | ﻿38811457  (95.6336%) | 2755711  (6.79023%) | 36055746  (88.8434%) |
| ﻿SCI-D56-moderate-1 | ﻿35997520 | ﻿35720440 | ﻿145.29 | ﻿5189755283 | ﻿96.90 | 91.78 | 46.73 | ﻿34172163  (95.6656%) | 2679110  (7.50022%) | 31493053  (88.1654%) |
| ﻿SCI-D56-moderate-2 | ﻿36594378 | ﻿36225260 | ﻿144.38 | ﻿5230224483 | ﻿96.71 | 91.31 | 44.33 | ﻿34687675  (95.7555%) | 3601054  (9.94073%) | 31086621  (85.8148%) |
| ﻿SCI-D56-moderate-3 | ﻿38836586 | ﻿38158506 | ﻿144.46 | ﻿5512278925 | ﻿96.86 | 91.69 | 45.48 | ﻿36492986  (95.6353%) | 2928086  (7.67348%) | 33564900  (87.9618%) |
| ﻿SCI-D56-severe-1 | ﻿41031364 | ﻿40691348 | ﻿145.05 | ﻿5902399770 | ﻿96.76 | 91.47 | 45.96 | ﻿38901584  (95.6016%) | 3203273  (7.87212%) | 35698311  (87.7295%) |
| ﻿SCI-D56-severe-2 | ﻿52151132 | ﻿51712740 | ﻿144.74 | ﻿7484658942 | ﻿96.97 | 91.93 | 45.88 | ﻿49537469  (95.7935%) | 3984922  (7.70588%) | 45552547  (88.0877%) |
| ﻿SCI-D56-severe-3 | ﻿49274472 | ﻿48880904 | ﻿145.11 | ﻿7093324812 | ﻿96.71 | 91.38 | 45.97 | ﻿46689971  (95.5178%) | 4177198  (8.54566%) | 42512773  (86.9721%) |

Note: 1. Sequencing was performed on an Illumina platform using paired-end 150 bp reads (PE150).

2.Mapping statistics (Total mapped, Uniquely mapped, Multiple mapped) are shown as both read counts and percentages of clean reads.
