# Supplementary material for: Establishment and multifaceted characterization of a graded spinal cord injury model based on graduated impact depth
Source: Animal Model Exp Med. 2026 Feb 27;9(4):809–21. doi: 10.1002/ame2.70150 (PMC13242734; doi:10.1002/ame2.70150)
Supplement: Supplementary file 8 — Data S1. [file AME2-9-809-s008.docx]

**Supplementary Methods**

**Histological staining**

For Hematoxylin–Eosin (HE) staining, the axial sections were deparaffinized with xylene, rehydrated through a graded ethanol series, and rinsed with distilled water. Hematoxylin staining was performed for 5 minutes, followed by a brief wash in distilled water. The sections were then differentiated in 1% acid ethanol for 10 seconds, thoroughly rinsed, and counterstained with eosin for 5 minutes before being subjected to another round of dehydration and clearing with xylene. Finally, neutral resin was applied to mount the sections. For Luxol Fast Blue (LFB) staining, rehydrated sections were immersed in LFB solution at 56°C overnight to label myelin. Excess stain was removed by rinsing with 95% ethanol, followed by differentiation using a lithium carbonate solution and 70% ethanol until the gray and white matter were clearly distinguishable under a microscope.

For immunofluorescence (IF) staining, antigen retrieval was performed by incubating the sagittal sections in sodium citrate buffer within a microwave for 10 minutes, followed by rinsing with 0.1 M PBS. Permeabilization was achieved using 0.5% Triton X-100 for 10 minutes, after which the sections were blocked with 5% goat serum at room temperature for 1 hour. Subsequently, sections were incubated overnight at 4°C with primary antibodies targeting GFAP (mouse anti-GFAP, 1:500, Proteintech), NeuN (rabbit anti-NeuN, 1:500, Abcam), IBA1 (rabbit anti IBA1, 1:500, Proteintech), and Olig2 (rabbit anti Olig2, 1:100, Proteintech) in a humidified chamber. Following three 10-minute washes in PBS, the sections were incubated with Alexa Fluor™ Plus 546-conjugated goat anti-mouse IgG (1:1000, ThermoFisher) and Alexa Fluor™ Plus 647-conjugated goat anti-rabbit IgG (1:1000, ThermoFisher) and for 1.5 hours at room temperature. After another series of PBS washes, the sections were stained with DAPI (1:1000, ThermoFisher) for 20 minutes and mounted using ProLong™ Gold Antifade Mountant (ThermoFisher). All stained sections were visualized using a slide scanner (VS200, Olympus), and images were analyzed using ImageJ software.

**Sample collection and RNA extraction**

Spinal cord tissues, including a 5 mm segment centered at the lesion site, were obtained from each group (sham, mild, moderate, and severe) at 1 dpi (acute), 3 dpi (subacute), 14 dpi (intermediate), and 56 dpi (chronic), respectively (n = 3 per group). Total RNA was extracted from the collected spinal cord using TRIzol reagent (Invitrogen). Subsequently, 1 μg total RNA was used for library preparation. The Poly(A) mRNA was isolated with Oligo (dT) beads, followed by mRNA fragmentation using divalent cations under high temperature conditions. Priming was performed using Random Primers. First strand cDNA and the second–strand cDNA were synthesized. The purified double-stranded cDNA underwent end-repair and dA-tailing in a single reaction, followed by a T–A ligation to attach adaptors at both ends. Adaptor-ligated DNA was then size-selected using DNA Clean Beads. Each sample was subjected to PCR amplification with P5 and P7 primers, and the resulting PCR products were validated. Finally, libraries with distinct indices were pooled and sequenced on an Illumina HiSeq platform using a 2×150 paired-end (PE) configuration in accordance with the manufacturer's protocol.

**Quality control and alignment of sequencing data**

To eliminate technical sequences, including adapters, polymerase chain reaction (PCR) primers, and fragments thereof, and quality of bases lower than 20, pass-filter data of fastq format were processed using Cutadapt (V1.9.1, phred cutoff: 20, error rate: 0.1, adapter overlap: 1bp, min. length: 75, proportion of N: 0.1) to be high quality clean data. For the alignment of sequencing data, reference genome sequences and gene model annotation files of relative species were downloaded from ENSEMBL. Next, the reference genome was indexed using Hisat2 (v2.2.1) to optimize alignment efficiency. Finally, the cleaned sequencing reads were mapped to the reference genome using Hisat2 (v2.2.1), ensuring accurate alignment for subsequent analysis.

**Gene expression and differential expression analysis**

﻿Gene expression levels were quantified by counting the number of reads mapped to each gene using HTSeq (v0.6.1). The expression values were then normalized and represented as Fragments Per Kilobase of transcript per Million mapped reads (FPKM). Differential expression analysis across groups, each consisting of three biological replicates, was conducted using the DESeq Bioconductor package. To minimize the false discovery rate (FDR), P-values obtained from DESeq analysis were adjusted following Benjamini and Hochberg’s correction method. Genes with an adjusted P-value below 0.05 were considered significantly differentially expressed (DEGs).
